# A Comparative Rugoscopic Study of the Dentate and Edentulous Individuals in the South Indian Population

**DOI:** 10.1155/2014/283428

**Published:** 2014-01-30

**Authors:** Jagdish Prasad Rajguru, Satya Ranjan Misra, Nagaveni S. Somayaji, K. M. K. Masthan, Aravindha N. Babu, Neeta Mohanty

**Affiliations:** ^1^Department of Oral Pathology, Hi-Tech Dental College, Pandara, Bhubaneswar, Odisha 751025, India; ^2^Department of Oral Medicine & Radiology, Institute of Dental Sciences, Kalinganagar, Bhubaneswar, Odisha 751003, India; ^3^Department of Prosthodontics, Hi-Tech Dental College, Pandara, Bhubaneswar, Odisha 751025, India; ^4^Department of Oral Pathology, Ragas Dental College, Uthandi, Chennai, Tamil Nadu 600096, India; ^5^Department of Oral Pathology, Institute of Dental Sciences, Kalinganagar, Bhubaneswar, Odisha 751003, India

## Abstract

This study analyzes the rugae pattern in dentulous and edentulous patients and also evaluates the association of rugae pattern between males and females. *Aims and Objectives*. This study aims to investigate rugae patterns in dentulous and edentulous patients of both sexes in South Indian population and to find whether palatoscopy is a useful tool in human identification. *Materials and Methods*. Four hundred outpatients from Sree Balaji Dental College and Hospital, Chennai, were included in the study. The study group was equally divided between the sexes, which was further categorized into 100 dentulous and edentulous patients, respectively. *Results*. The edentulous male showed the highest mean of wavy pattern and total absence of circular pattern while the edentulous female group showed the highest mean of curved pattern and total absence of nonspecific pattern, while dentate population showed similar value as that of the overall population such as straight, wavy, and curved patterns. *Conclusion*. The present study concludes that there is similar rugae pattern of distribution between male and female dentate population while there is varied pattern between the sexes of edentulous population. However, the most predominant patterns were straight, wavy, and circular patterns.

## 1. Introduction

Human identification is one of the most challenging subjects that we have been confronted with since ages. Palatal rugae, also called as plicae palatinae transversae or rugae palatine, refers to the ridges or elevations on the anterior part of the palatal mucosa present on either side of the mid palatine raphe behind the incisive papilla. It is widely present in mammals. Study of the palatal rugae is known as rugoscopy or palatoscopy, and it can be successfully implemented to reveal the identity of an individual. Rugoscopy can be used as compensatory process to dactyloscopy. Palatal rugae had been applied in various fields such as anthropology, comparative anatomy, genetics, forensic odontology, prosthodontics, and orthodontics.

Palatine rugae are irregular, asymmetric ridges of mucous membrane extending laterally from the incisive papilla and the anterior part of the median palatal raphe. There are approximately four rugae on each side of the palate. Slightly more rugae are found in males and on the left side in both genders. Generally, there is no bilateral symmetry in the number of primary rugae or in their angulations from the midline [1]. Catastrophic accidents involving plane crashes, fires, and explosions can destroy fingerprints, but interestingly palatal rugae patterns are preserved [2]. Kuppler, 1897, was the first person to study the palatal anatomy for identifying various races. However, the term rugoscopy (palatoscopy) was first proposed by Trobo Hermosa in 1932. There are various classifications of palatine rugae based on their form, position, shape, size, direction, unification of the rugae, and a person's personality [3]. This study aims to investigate rugae patterns in dentulous and edentulous patients of both sexes.

## 2. Materials and Methods

Four hundred outpatients from Sree Balaji Dental College and Hospital were included in the study. The study group was equally divided between the sexes, which was further categorized into 100 dentulous and edentulous patients, respectively. The edentulous patients who participated in the study were not using dentures. The primary impressions were made using alginate impression material and the study cast was prepared with dental stone (Type-3 Gypsum products). It was air-dried in natural sunlight and was prepared for analytical procedure. Under daylight a magnifying lens was used to imprint palatal rugae from the study model. The different morphological rugae patterns appreciated were circular, straight, wavy, curve, unification, and nonspecific. Data was analyzed using SPSS system. Student's *t*-test analysis was done and *P* value was determined.

## 3. Result

The rugae pattern of four hundred individuals was evaluated. When the mean of male and female rugae was analyzed irrespective of their dental status, the predominant patterns were wavy, curve, and straight. Males showed wavy pattern followed by curve and straight, while females showed a predominance of curve and equal ratio of straight and wavy patterns. When the patterns between the sexes were analyzed by *t*-test, there was statistically significant difference in straight (*P* < 0.001), wavy (*P* < 0.001), circular (*P* = 0.058), and nonspecific (*P* = 0.001) patterns. The mean values of dentulous and edentulous patients were compared irrespective of their gender; wavy, curve, and straight patterns were predominant in both groups. When the patterns were analyzed between the dentate and nondentate population there existed a significant difference in the order of straight (*P* < 0.001), curve (*P* < 0.001), wavy (*P* < 0.001), circular (*P* = 0.058), unification (*P* < 0.001), and nonspecific (*P* = 0.001) patterns.

Considering the dental status in the sexes, the dentate males and females showed a statistically significant difference in nonspecific (*P* = 0.048), straight (*P* = 0.001), and wavy (*P* = 0.001) patterns, while the edentulous group showed significant difference in straight (*P* = 0.020), wavy (*P* < 0.001), unification (*P* < 0.001), nonspecific (*P* = 0.001), and circular (*P* = 0.048) patterns.

When the mean of individual rugae patterns was compared between the sexes and the dental status the edentulous male group showed the highest mean of wavy pattern and total absence of circular pattern while the edentulous female group showed the highest mean of curved pattern and total absence of nonspecific pattern, while dentate population showed similar value as that of the overall population such as straight, wavy, and curved patterns (see Tables 1, 2, 3 and 4).

## 4. Discussion

The palatal rugae are significant for an individual as they are said to remain unchanged during one's lifetime [4]. However, some events can contribute to changes in rugae pattern, including extreme finger sucking in infancy and persistent pressure due to orthodontic treatment [5]. In fact, palatal rugae stability is considered an important factor when teeth are extracted. In humans they are symmetrical, which is an exclusive feature of human beings [1, 6].

According to English's studies [7], palatal rugae patterns are sufficiently characteristic to discriminate between individuals. In fact, these authors found it legitimate to base identification upon their comparison [7], allowing for human identification even in extreme circumstances [8] (see Figures 4 and 5). Furthermore, the ability of palatal rugae to resist decomposition changes for up to seven days after death was also noted [9].

Our study reveals that there is similar pattern of distribution between male and female dentate population while there is varied pattern between the sexes of edentulous population. However, the most predominant patterns were straight, wavy, and circular patterns. There exists a statistically significant difference between some of the patterns between the sexes and they may provide a valuable tool in identifying the gender.

## 5. Various Methods of Palatoscopy

Intraoral inspection, study of models, calcorrugoscopy or overlay print, stereoscopy, and stereophotogrammetry. There is however considerable debate on this matter [10, 11].

## 6. Limitations of Rugoscopy

**Figure 1:**
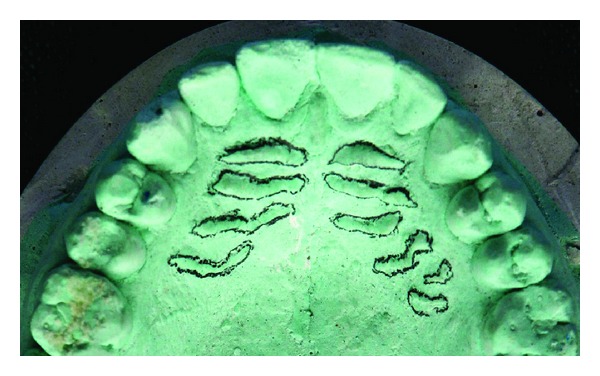
Photograph showing highlighted palatal rugae on maxillary dentulous cast.

**Figure 2:**
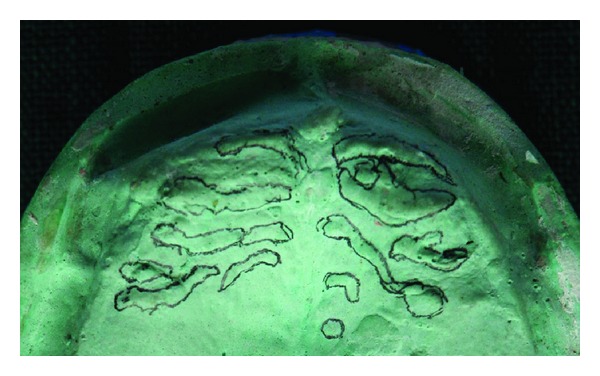
Photograph showing highlighted palatal rugae on maxillary edentulous cast.

**Figure 3:**
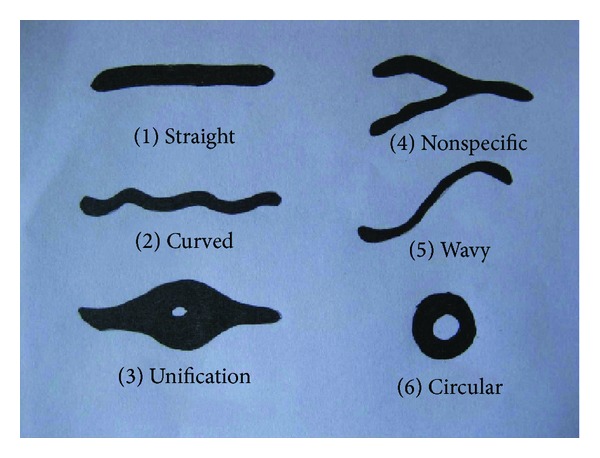
Line diagrams showing various patterns of palatal rugae in humans.

**Figure 4:**
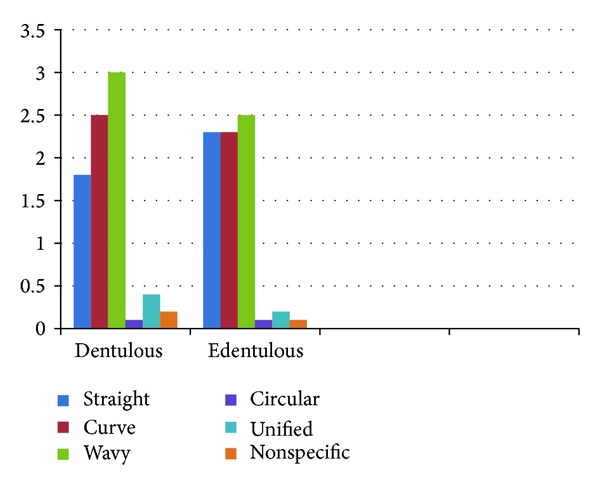
Comparison of rugae patterns among dentulous and edentulous subjects.

**Figure 5:**
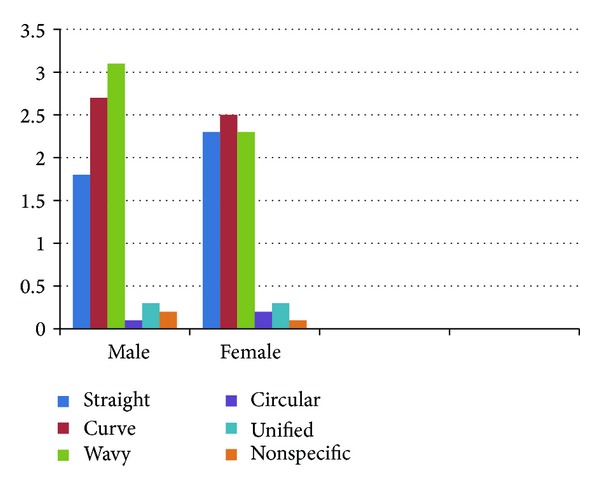
Comparison of rugae patterns among males and females.

As this method is not the primary source of forensic investigation, this can be utilized as an adjunctive source for human identification. In fact, contrary to lip prints, it is possible to have antemortem data in different forms (dental casts, old prosthetic maxillary appliances, and intraoral photographs) (see Figures 1, 2, and 3). However, pictures might not be so useful in decomposed body in crime investigation. One more limitation of palatal rugae is possibility of rugae pattern forgery [3, 12]. Investigators aim to assess its identification. Characteristics of palatal rugae pattern did not change as a result of growth and remained stable from the time of development until mucosa is degenerated by death.

## 7. Conclusion

Though rugoscopy in forensic sciences cannot be equated to fingerprinting it is considered as an important complementary method in identification of a person. Studies related to the ethnicity are available in the literature; however, our study aimed at differentiating the gender. Though there are statistically significant differences between the sexes, a study with large group including the samples from various other countries is mandatory, thereby establishing a wide application of rugoscopy.

## Figures and Tables

**Table 1:** Table showing the mean value of different rugae patterns among edentulous subjects.

	Male edentulous	Female edentulous	
Straight	2.20	2.50	0.020*
Curve	2.40	2.30	0.488
Wavy	0.00	0.10	<0.001*
Circular	0.10	0.05	0.048
Unification	0.20	0.10	<0.001*
Nonspecific	0.10	0.00	0.001*

*Statistically significant value.

**Table 2:** Table showing the mean value of different rugae patterns among dentulous subjects.

	Male dentulous	Female dentulous	
Straight	1.40	2.10	0.001*
Curve	3.00	2.80	0.197
Wavy	3.30	2.60	<0.001*
Circular	0.10	0.10	1.000
Unification	0.40	0.50	0.342
Nonspecific	0.20	0.10	0.048

*Statistically significant value.

**Table 3:** Table showing the mean value and statistically significant *P* value in different rugae patterns of dentulous and edentulous subjects.

	Dentulous	Edentulous	
Straight	1.75	2.35	<0.001*
Curve	2.90	2.35	<0.001*
Wavy	2.95	2.50	<0.001*
Circular	0.10	0.05	0.058
Unification	0.45	0.15	<0.001*
Nonspecific	0.15	0.05	0.001*

*Statistically significant value.

**Table 4:** Table showing comparison of rugae patterns among males and females of dentulous and edentulous subjects.

	Male	Female	
STRAIGHT	1.80	2.30	<0.001*
CURVE	2.70	2.55	0.169
WAVY	3.10	2.35	<0.001*
CIRCULAR	0.05	0.10	0.058
UNIFICATION	0.30	0.30	1.000
NONSPECIFIC	0.15	0.05	0.001*

*Statistically significant value.
